# Biotechnological Drugs: The Breakthrough in Autoimmune Rheumatic Conditions

**DOI:** 10.1155/2014/481973

**Published:** 2014-08-10

**Authors:** Lorenzo Cavagna, Lesley-Ann Saketkoo, Andreas Schwarting, Roberto Caporali

**Affiliations:** ^1^Division of Rheumatology, University and IRCCS Policlinico S. Matteo Foundation, Viale Golgi 2, 27100 Pavia, Italy; ^2^Sections of Rheumatology and Pulmonary Medicine, Department of Medicine, Louisiana State University Health Sciences Center, 1542 Tulane Avenue, New Orleans, LA, USA; ^3^Division of Rheumatology, Mainz University Hospital and ACURA Rheumatology Center, Kaiser-Wilhelm Street 9-11, Rhineland-Palatinate, 55543 Bad Kreuznach, Germany

Biotechnological drugs are a wide group of therapeutic agents obtained by means of genetic engineering methodologies. They act through the inhibition of specific cytokines/cells involved in the cascade of different pathological processes, leading to the modification of host immune response.

The main targets of these drugs are TNF-*α*, IL-6, IL-1, and B and T cells. In the latest years, according to the improved knowledge in the pathogenesis of several diseases, the use of these drugs is steadily increasing, and new agents have been marketed or are under development.

Autoimmune rheumatic diseases are the prototypical example of pathological conditions that may benefit from these therapies. Biotechnological drugs deeply improved patients' prognosis and quality of life in several rheumatic conditions, as, for example, rheumatoid arthritis (RA). Despite the large number of papers published and the large clinical experience obtained for some of these drugs, there are still many questions that should be clarified. This special issue is addressed to the analysis of some peculiar aspects of these drugs, in both clinic and research context.

F. Atzeni et al. evaluated in a real life study the effects of anti-TNF-*α* drugs on RA related disability, suggesting that benefits from these therapies may be relevant also in patients with long-standing diseases. Interestingly, results showed that the improvement in disability was marked during the first year of anti-TNF therapy, with a subsequent slower but significant recovery over the subsequent four years.

The article by L. Cavagna and W. J. Taylor is focused on gout: this disease is the typical example of how, in few years, technologic advances improved therapeutic approach. New and old biologics on pipeline are extensively reviewed, in order to help clinicians in their daily activity.

E. G. Favalli et al. managed an interesting review addressed to RA therapeutic approach: they demonstrate that, with respect to treatments available, up to now only a limited number of head-to-head randomized controlled trials are available, with subsequent limitations in the optimal use of different drugs available in daily practice. However, the authors provided some preliminary but encouraging suggestions on how to deal with the complexity of the available therapeutic armamentarium.

M. Schulz et al. assessed the differences in TNF-*α* regulation in patients with ankylosing spondylitis (AS) and RA being treated with infliximab and etanercept. Results suggested that drug metabolism between these diseases is different and they are a further input for the pathogenetic studies in both conditions.

The paper of F. Genre et al. deeply analysed the role of adipokines in the assessment of cardiovascular risk of AS, particularly in anti-TNF-*α* treated patients. By considering the impact of cardiovascular mortality in the setting, authors' suggestions are an important impulse for further studies on these interesting molecules.

Finally, Y. Hu et al. first analysed the role of a peculiar neuronal nicotinic acetylcholine receptor, named *α*7nAChR, and of its partial agonist GTS-21 in the experimental model of collagen induced arthritis (CIA), identifying another interesting and potential target of treatment in RA.

*Lorenzo Cavagna* *Lesley-Ann Saketkoo* *Andreas Schwarting* *Roberto Caporali*

